# Essential role of microglial transforming growth factor-β1 in antidepressant actions of (*R*)-ketamine and the novel antidepressant TGF-β1

**DOI:** 10.1038/s41398-020-0733-x

**Published:** 2020-01-27

**Authors:** Kai Zhang, Chun Yang, Lijia Chang, Akemi Sakamoto, Toru Suzuki, Yuko Fujita, Youge Qu, Siming Wang, Yaoyu Pu, Yunfei Tan, Xingming Wang, Tamaki Ishima, Yukihiko Shirayama, Masahiko Hatano, Kenji F. Tanaka, Kenji Hashimoto

**Affiliations:** 1 Division of Clinical Neuroscience, Chiba University Center for Forensic Mental Health, Chiba, 260-8670 Japan; 2 Department of Biomedical Science, Chiba University Graduate School of Medicine, Chiba, 260-8670 Japan; 3 Department of Neuropsychiatry, Keio University School of Medicine, Tokyo, 160-8585 Japan; 4 Department of Psychiatry, Teikyo University Chiba Medical Center, Chiba, 299-0111 Japan; 5 Present Address: Department of Psychiatry, Chaohu Hospital of Anhui Medical University, Hefei, 238000 China; 6 Present Address: Department of Anesthesiology and Perioperative Medicine, The First Affiliated Hospital of Nanjing Medical University, Nanjing, 210029 China

**Keywords:** Depression, Clinical pharmacology

## Abstract

In rodent models of depression, (*R*)-ketamine has greater potency and longer-lasting antidepressant effects than (*S*)-ketamine; however, the precise molecular mechanisms underlying the antidepressant actions of (*R*)-ketamine remain unknown. Using RNA-sequencing analysis, we identified novel molecular targets that contribute to the different antidepressant effects of the two enantiomers. Either (*R*)-ketamine (10 mg/kg) or (*S*)-ketamine (10 mg/kg) was administered to susceptible mice after chronic social defeat stress (CSDS). RNA-sequencing analysis of prefrontal cortex (PFC) and subsequent GSEA (gene set enrichment analysis) revealed that transforming growth factor (TGF)-β signaling might contribute to the different antidepressant effects of the two enantiomers. (*R*)-ketamine, but not (*S*)-ketamine, ameliorated the reduced expressions of *Tgfb1* and its receptors (*Tgfbr1* and *Tgfbr2*) in the PFC and hippocampus of CSDS susceptible mice. Either pharmacological inhibitors (i.e., RepSox and SB431542) or neutralizing antibody of TGF-β1 blocked the antidepressant effects of (*R*)-ketamine in CSDS susceptible mice. Moreover, depletion of microglia by the colony-stimulating factor 1 receptor (CSF1R) inhibitor PLX3397 blocked the antidepressant effects of (*R*)-ketamine in CSDS susceptible mice. Similar to (*R*)-ketamine, the recombinant TGF-β1 elicited rapid and long-lasting antidepressant effects in animal models of depression. Our data implicate a novel microglial TGF-β1-dependent mechanism underlying the antidepressant effects of (*R*)-ketamine in rodents with depression-like phenotype. Moreover, TGF-β1 and its receptor agonists would likely constitute a novel rapid-acting and sustained antidepressant in humans.

## Introduction

In 1990, Trullas and Skolnick^1^ demonstrated that *N*-methyl-D-aspartate receptor (NMDAR) antagonists such as (+)-MK-801 showed antidepressant-like effects in rodents. In 2000, Berman et al.^2^ demonstrated the rapid-acting and sustained antidepressant effects of the NMDAR antagonist ketamine in patients with major depressive disorder (MDD). Subsequently, several groups replicated the robust antidepressant effects of ketamine in treatment-resistant patients with either MDD or bipolar disorder^3–10^. Interestingly, ketamine rapidly reduced suicidal thoughts in depressed patients with suicidal ideation within 1 day and for up to 1 week^11,12^. In addition, it is suggested that suicidal thoughts may be related to symptoms of anhedonia independent of other depressive symptoms^13^. Meta-analyses revealed that ketamine has rapid-acting and sustained antidepressant effects and anti-suicidal ideation effects in treatment-resistant patients with depression^14–16^. Importantly, meta-analyses showed that the effect sizes of ketamine are larger than those of other NMDAR antagonists^14,15^, suggesting that NMDAR blockade is not a sole mechanism of antidepressant action for ketamine. The collective rapid-acting and sustained antidepressant actions of ketamine in depressed patients are serendipitous in the field of psychiatry;^17–19^ however, the precise molecular and cellular mechanisms underlying antidepressant effects of ketamine remain to be elucidated^20–25^. Off-label use of ketamine is popular in the United States (US), although the adverse side-effects (i.e., psychotomimetic effects, dissociation, and abuse liability) of ketamine remain to be resolved^26,27^.

Ketamine (Ki = 0.53 μM for NMDAR), also known as (*R,S*)-ketamine, is a racemic mixture that contains equal amounts of (*R*)-ketamine (or arketamine) (Ki = 1.4 μM for NMDAR) and (*S*)-ketamine (or esketamine) (Ki = 0.30 μM for NMDAR)^24^. Preclinical data have shown that (*R*)-ketamine displays greater potency and longer -lasting antidepressant effects than (*S*)-ketamine in rodent models of depression^28–34^, suggesting that NMDARs do not play a major role in the robust antidepressant effects of ketamine^24^. Importantly, in both rodents and monkey, the side-effects of (*R*)-ketamine were lower than were those of (*R,S*)-ketamine and (*S*)-ketamine^29,35–38^. In addition, in humans, the incidence of psychotomimetic side-effects of (*S*)-ketamine (0.45 mg/kg) was higher than that of (*R*)-ketamine (1.8 mg/kg), although the dose of (*S*)-ketamine was lower than was that of (*R*)-ketamine^39^. Though (*S*)-ketamine produced psychotic reactions, including depersonalization and hallucinations, the same dosage of (*R*)-ketamine did not induce psychotic symptoms in the healthy subjects, and most of them experienced a state of relaxation^40^. These results indicate that (*S*)-ketamine contributes to the acute side-effects of ketamine, whereas (*R*)-ketamine may not be associated with these side-effects^22,24^. On 5 March 2019, the US Food & Drug Administration approved (*S*)-ketamine nasal spray for treatment-resistant depressed patients. Due to the risk of serious adverse effects, (*S*)-ketamine nasal spray can be obtained only through a restricted distribution system under the Risk Evaluation and Mitigation Strategy. A clinical trial of (*R*)-ketamine in humans is underway^24^. Meanwhile, little is known about the precise molecular mechanisms underlying the different antidepressant effects of the two enantiomers^24,25,41,42^.

The aim of this study was to identify the novel molecular mechanisms underlying the antidepressant effects of (*R*)-ketamine in animal models of depression. First, we conducted RNA-sequencing analysis of the prefrontal cortex (PFC) of chronic social defeat stress (CSDS) susceptible mice treated with either (*R*)-ketamine or (*S*)-ketamine, as PFC contributes to the antidepressant actions of ketamine and its enantiomers^29,43,44^. Second, we studied the effects of pharmacological inhibitors and a neutralizing antibody of the novel target in the antidepressant effects of (*R*)-ketamine. Finally, we investigated whether the novel molecule (i.e., TGF-β) has rapid-acting and sustained antidepressant effects in rodent models of depression.

## Materials and methods

### Animals

Male adult C57BL/6 mice, aged 8 weeks (body weight 20–25 g, Japan SLC, Inc., Hamamatsu, Japan), male CD1 mice, aged 14 weeks (body weight 40–45 g, Japan SLC, Inc., Hamamatsu, Japan) were used in the experiments. Male Sprague-Dawley rats, aged 7 weeks (body weight 200–230 g, Charles-River Japan, Co., Tokyo, Japan) were used for learned helplessness (LH) model. No blinding for animal experiments was done. Animals were housed under controlled temperature and 12 h light/dark cycles (lights on between 07:00–19:00), with ad libitum food and water. The study was approved by the Chiba University Institutional Animal Care and Use Committee.

### Compounds and treatment

(*R*)-ketamine hydrochloride and (*S*)-ketamine hydrochloride were prepared by recrystallization of (*R,S*)-ketamine (Ketalar^®^, ketamine hydrochloride, Daiichi Sankyo Pharmaceutical Ltd., Tokyo, Japan) and D-(-)-tartaric acid (or L− (+)-tartaric acid), respectively^28^. The purity of these enantiomers was determined by a high-performance liquid chromatography (CHIRALPAK^®^ IA, Column size: 250 × 4.6 mm, Mobile phase: n-hexane/dichloromethane/diethylamine (75/25/0.1), Daicel Corporation, Tokyo, Japan)^28^. The dose (10 mg/kg as hydrochloride salt) of (*R*)-ketamine and (*S*)-ketamine was selected as reported previously^28,29,32–35^. RepSox (10 mg/kg, i.p., a TGF-β1 receptor inhibitor; Selleck Chemicals, Co., Ltd, Houston, TX, USA), SB431542 (10 μM, 2 μl, i.c.v., a TGF-β1 receptor inhibitor; Tocris Bioscience, Ltd., Bristol, UK), neutralized TGF-β antibody (Catalog #: MAB1835–500; R&D System, Inc. Minneapolis, MN), and mouse IgG1 control antibody (Catalog #: MAB002; R&D System, Inc. Minneapolis, MN) were used. Recombinant mouse TGF-β1 (Catalog #: 7666-MB-005; R&D System, Inc. Minneapolis, MN) and recombinant mouse TGF-β2 (Catalog #: 302-B2; R&D System, Inc. Minneapolis, MN) were used as previously reported^45,46^. PLX3397 [Pexidartinib: a colony-stimulating factor 1 receptor (CSF1R) inhibitor, MedChemExpress Co., Ltd., Monmouth Junction, NJ] was used to decrease microglia in the brain. LPS (Catalog #: L-4130, serotype 0111:B4, Sigma-Aldrich, St Louis, MO, USA) was used for inflammation model of depression. Other reagents were purchased commercially.

### CSDS model and LPS-induced model

The procedure of CSDS was performed as previously reported^29,32–34,47^. Detailed methods were shown in the supplemental information.

### RNA-sequencing analysis

**Fig. 1:**
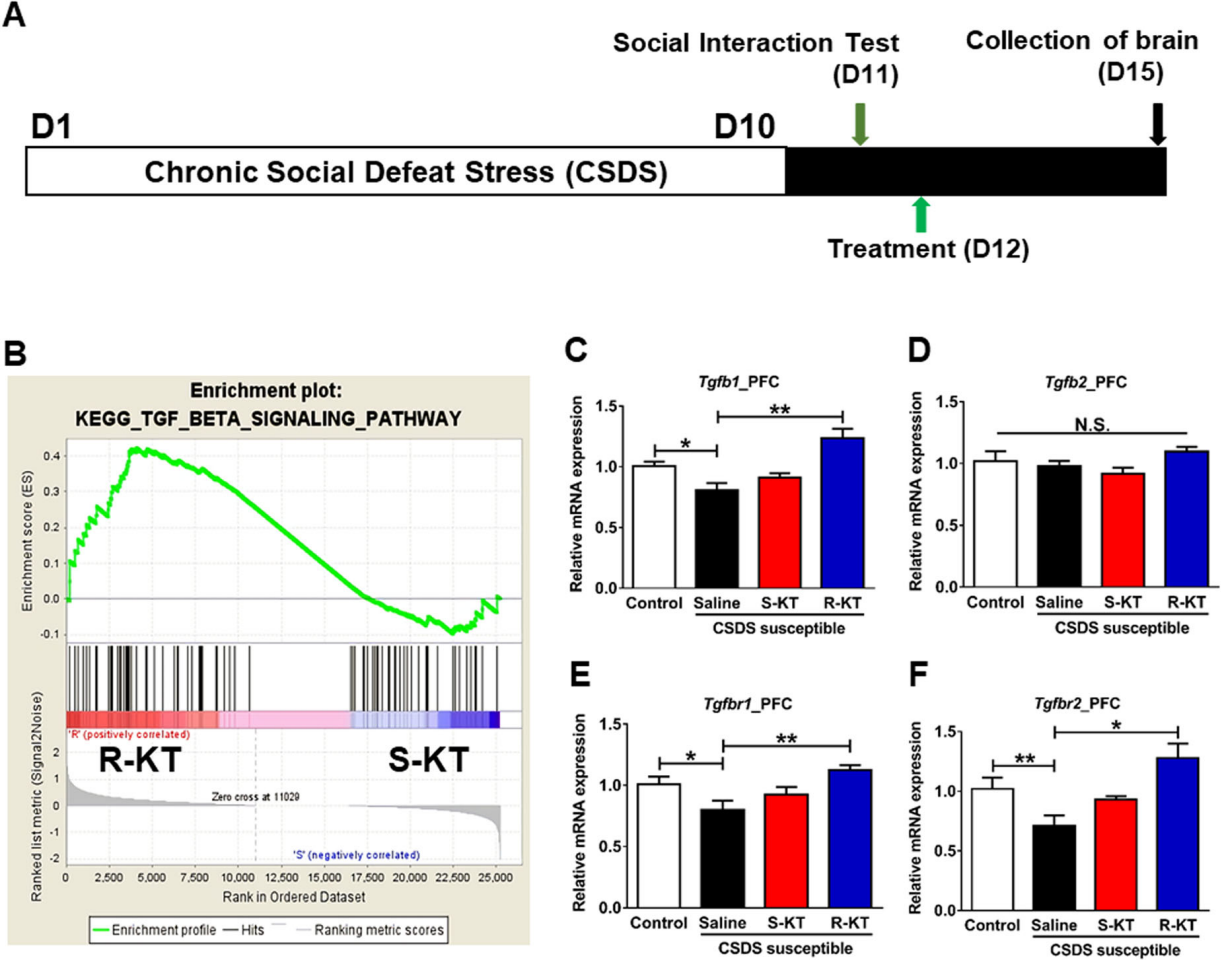
Schedule of CSDS, treatment, RNA-sequencing analysis, and gene expression. **a** The schedule of chronic social defeat stress (CSDS) model, treatment, and collection of brain. **b** GSEA: TGF-β signaling. **c** *Tgfb1* mRNA in the PFC (one-way ANOVA, *F*_3,20_ = 10.827, *P* < 0.001). **d** *Tgfb2* mRNA in the PFC (one-way ANOVA, *F*_3,20_ = 1.795, *P* = 0.181). **e** *Tgfbr1* mRNA in the PFC (one-way ANOVA, *F*_3,20_ = 5.175, *P* = 0.008). **f** *Tgfbr2* mRNA in the PFC (one-way ANOVA, *F*_3,20_ = 6.801, *P* = 0.002). Data are shown as mean ± SEM. (*n* = 6). *P < 0.05, **P < 0.01. ANOVA, analysis of variance; GSEA gene set enrichment analysis, N.S. not significant, R-KT (*R*)-ketamine, S-KT (*S*)-ketamine.

(*R*)-Ketamine (10 mg/kg) or (*S*)-ketamine (10 mg/kg) was administered intraperitoneally (i.p.) to susceptible mice after CSDS (Fig. 1a). PFC was collected 3 days after a single administration. RNA-sequencing analysis of PFC samples was performed at Tataka Bio Inc. (Kusatsu, Shiga, Japan). Analysis of the biological functions was performed using gene set enrichment analysis (GSEA)(http://software.broadinstitute.org/gsea/index.jsp).

### Gene expression analysis by quantitative real-time PCR

Control mice and CSDS susceptible mice were sacrificed 3 days after intraperitoneal (i.p.) administration of saline (10 ml/kg), (*R*)-ketamine (10 mg/kg), or (S)-ketamine (10 mg/kg). The PFC and hippocampus were quickly dissected on ice from whole brain since these brain regions play a key role in antidepressant effects of (*R*)-ketamine^44^. Detailed methods were shown in the supplemental information.

### Inhibition of TGF-β1 inhibitors and neutralizing antibody

To examine the role of TGF-β1 in the antidepressant effects of (*R*)-ketamine, two inhibitors (RepSox and SB431542) of TGF-β receptor 1 were used. RepSox (10 mg/kg, i.p.) or vehicle (10 ml/kg, i.p.) was injected 30 min before i.p. administration of (*R*)-ketamine (10 mg/kg) in CSDS susceptible mice. SB431542 (10 μM, 2 μl, i.c.v.) or vehicle (2 μl, i.c.v.) was injected 30 min before i.p. administration of (*R*)-ketamine (10 mg/kg) in CSDS susceptible mice. The neutralizing antibody of TGF-β1 (1 μg/ml, 2 μL, i.c.v.) or control antibody (1 μg/ml, 2 μL, i.c.v.) was injected 30 min before i.p. administration of (*R*)-ketamine (10 mg/kg) in CSDS susceptible mice. Subsequently, behavioral tests were performed.

### Depletion of microglia by PLX3397

PLX3397 was reported to eliminate microglia in the brain^48–50^. For preliminary experiment, PLX3397 (10 μM or 100 μM, 2 μl, i.c.v.) or vehicle [10% dimethyl sulfoxide (DMSO) and 90% (sulfobutylether-β-cyclodextrin)(SBE-β-CD)] was administered to mice under isoflurane anesthesia. The PFC was collected 6, 12, and 24 h after i.c.v. infusion, and Western blot analysis of Iba1 in the PFC was performed.

To examine the effects of microglia depletion, PLX3397 (100 μM, 2 μl, i.c.v.) or vehicle (10% DMSO and 90% SBE-β-CD) was administered to mice under isoflurane anesthesia. PFC was collected 24 h after injection. Right PFC and left PFC were used for FACS analysis and Western blot of Iba1, respectively.

To examine the effects of microglia depletion on antidepressant effects of (*R*)-ketamine, PLX3397 (100 μM, 2 μl, i.c.v.) or vehicle (10% DMSO and 90% SBE-β-CD) was administered to CSDS susceptible mice under isoflurane anesthesia. Saline (10 ml/kg) or (*R*)-ketamine (10 mg/kg) was administered i.p. 24 h after injection of PLX3397 or vehicle. Subsequently, behavioral tests were performed.

### Antidepressant effects of TGF-β1 in a CSDS model

Effects of recombinant TGF-β1 in a CSDS model, LPS model, and LH model were examined. Saline (2 μL, i.c.v.) or (*R*)-ketamine (1 mg/ml, 2 μL, i.c.v.) was administered to CSDS susceptible mice. Saline (2 μL, i.c.v.) was administered to control mice. Subsequently, behavioral tests were performed.

### Antidepressant effects of TGF-β1 in a LPS-induced inflammation model

Inflammation model by lipopolysaccharide (LPS) was performed as previously reported^51–53^. Saline (10 ml/kg) or LPS (0.5 mg/kg) was administered i.p. to mice. Under isoflurane anesthesia, saline (2 μl, i.c.v.) or TGF-β1 (10 ng/μl, 2 μl, i.c.v.) was administered to mice 23 hrs after LPS administration. The locomotion and FST were performed 1 and 3 h after injection, respectively.

For intranasal administration, saline (15 μl) or TGF-β1 (1.5 μg, 15 μl) was administered to mice 23 hrs after LPS administration, as previously reported^38^. Mice were restrained by hand, and saline or TGF-β1 was administered intranasally into awake mice using Eppendorf micropipette (Eppendorf Japan, Tokyo, Japan). The locomotion and FST were performed 1 and 3 h after injection, respectively.

### Behavioral tests

Behavioral tests including locomotion, tail suspension test (TST), forced swimming test (FST), and one % sucrose preference test (SPT) were performed as previously reported^28,29,32–34^. Detailed methods were shown in the supplemental information.

### Learned helplessness (LH) model

Rat LH paradigm was performed as previously reported^44,54^. Detailed methods were shown in the supplemental information.

### Western blot analysis of Iba1

Western blot analysis was performed as reported previously^29,34,51,52^. Detailed methods were shown in the supplemental information.

### Double staining by in situ hybridization and immunohistochemistry

Mice were deeply anesthetized with isoflurane and sodium pentobarbital, and transcardially perfused with 4% paraformaldehyde in 0.1 M phosphate buffer (pH 7.4). The brains were further immersed in the same fixative overnight, cryoprotected in 20% sucrose/phosphate-buffered saline (PBS), and frozen by liquid nitrogen. The brains were sectioned coronally on a cryostat (CM3050S; Leica Biosystems, Germany) at 25 μm thickness. The cryosections were treated with proteinase K (40 μg/ml; Merck). After they were washed and acetylated, sections were incubated with a digoxigenin (DIG)-labeled mouse *Tgfb1* (cat#: G430055L01), *Tgfbr1* (cat#: F630025J19), or *Tgfbr2* (cat#: I420016D17) cRNA probes (DNAFORM, Yokohama, Kanagawa, Japan). After the sections were washed in buffers with serial differences in stringency, they were incubated with an alkaline phosphatase-conjugated anti-DIG antibody (1:5000; Roche, Japan). The cRNA probes were visualized with freshly prepared colorimetric substrate (NBT/BCIP; Roche, Japan). After visualized, sections were incubated with primary antibodies overnight at RT. All antibodies were diluted in PBS with 0.1% Triton X-100. The following antibodies were used: anti-Iba1 (cat#: 019–19741, 1:1000, rabbit, polyclonal; Wako, Japan), and anti-S100b (cat#: ab52642, 1:200, rabbit, monoclonal; Abcam, Cambridge, UK). the sections were sequentially incubated with anti-rabbit IgG biotinylated secondary antibodies (1:250, goat, polyclonal; Vector Laboratories, USA) for 90 min at room temperature (RT), an avidin-biotin complex (Vector Laboratories, USA) for 30 min at RT, and then the colorimetric reactions were developed with DAB (3,3′-diaminobenzidine) (ImmPACT DAB; Vector Laboratories, USA). Images of the sections were captured using a light microscope (BZ-X710; Keyence, Japan).

### FACS analysis

Mouse PFC tissues were mashed and passed through a 70 μm mesh to prepare single cell suspension then subjected for FACS analysis. Cells were stained with monoclonal antibodies against cell surface antigens at 4 °C for 30 min, then washed with PBS. In indicated cells, cells were fixed and permeabilized using FoxP3 staining buffer set (Invitrogen) according to the manufacturer instruction. Then intracellular antigens were stained with indicated antibodies at room temperature for 30 min. The following antibodies were used for staining; anti TMEM119-PE (Abcam, Cambridge, UK), allophycocyanin conjugated anti CD11b (BD Bioscience, Franklin Lakes, NJ), anti Iba1-FITC (Abcam), anti TGF-β-allophycocyanin (BioLegend, San Diego, CA). The stained cells were analyzed using FACSCantII and FlowJo software (BD).

### Statistical analysis

The data show as the mean ± standard error of the mean (S.E.M.). Analysis was performed using PASW Statistics 20 (formerly SPSS Statistics; SPSS). The data were analyzed using Student *t*-test or the one-way analysis of variance (ANOVA), followed by post hoc Tukey test. The *P*-values < 0.05 were considered statistically significant.

## Results

### RNA-sequencing analysis of PFC samples

To identify the novel molecular targets for the antidepressant effects of (*R*)-ketamine, we collected PFC samples 3 days after either (*R*)-ketamine (10 mg/kg) or (*S*)-ketamine (10 mg/kg) were administered to CSDS susceptible mice. We performed RNA-sequencing analysis of PFC samples from animals treated with either (*R*)-ketamine or (*S*)-ketamine (Fig. 1a). GSEA revealed that TGF-β signaling might be involved in the differential antidepressant effects of the two enantiomers (Fig. 1b). We found reduced expression of *Tgfb1* and its receptors (*Tgfbr1* and *Tgfbr2*) in the PFC and hippocampus from CSDS susceptible mice (Fig. 1c–f**and** Fig. S1). Conversely, the expression of *Tgfb2* in the PFC and the hippocampus did not differ in the four groups (Fig. 1c–f and Fig. S1). Interestingly, (*R*)-ketamine (10 mg/kg), but not (*S*)-ketamine (10 mg/kg), significantly ameliorated the reduced expression of these genes (Fig. 1c–f and Fig. S1).

### Effects of TGF-β1 inhibitors and neutralizing antibody in the antidepressant effects of (*R*)-ketamine

**Fig. 2:**
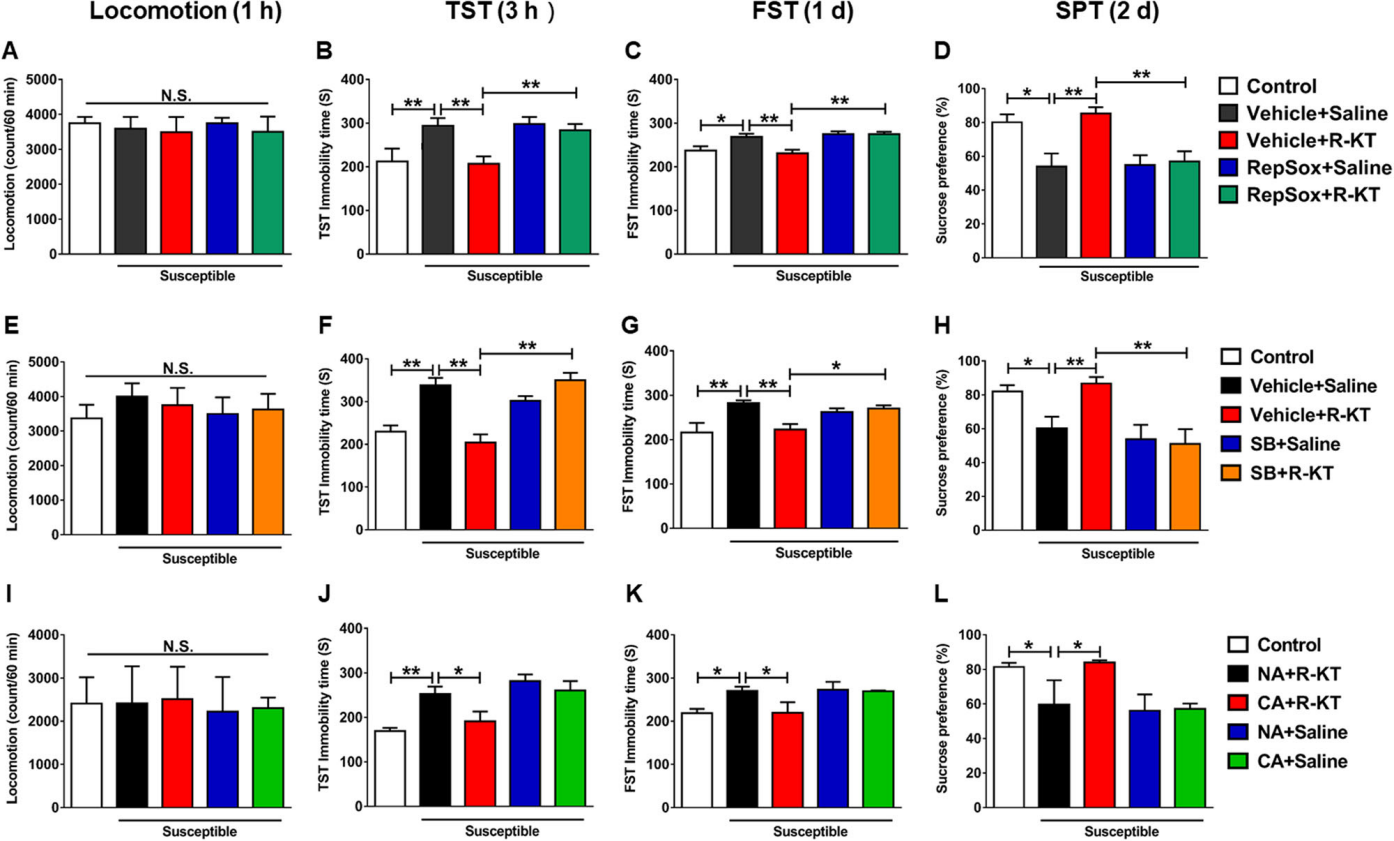
Effects of TGF-β1 inhibitors (RepSox and SB431542) and neutralizing TGF-β1 antibody on antidepressant effects of (*R*)-ketamine in CSDS model. **a** Locomotion (1 h, one-way ANOVA, *F*_4,35_ = 0.146, *P* = 0.964). **b** TST (3 h, one-way ANOVA, *F*_4,35 _ = 5.439, *P* = 0.002). **c** FST (1 day, one-way ANOVA, *F*_4,35_ = 2.919, *P* = 0.035). **d** SPT (2 days, one-way ANOVA, *F*_4,35_ = 7.011, *P* < 0.001). Data are shown as mean ± SEM. (*n* = 8). *P < 0.05, **P < 0.01. **e** Locomotion (1 h, one-way ANOVA, *F*_4,35_ = 0.299, *P* = 0.877). **f** TST (3 h, one-way ANOVA, *F*_4,35_ = 16.586, *P* < 0.001). **g** FST (1 day, one-way ANOVA, *F*_4,35_ = 4.686, *P* = 0.004). **h** SPT (2 day, one-way ANOVA, *F*_4,35_ = 6.161, *P* = 0.001). **i** Locomotion (1 h, one-way ANOVA, *F*_4,25_ = 0.020, *P* = 0.999). **j** TST (3 h, one-way ANOVA, *F*_4,35 _ = 8.165, *P* < 0.001). **k** FST (1 day, one-way ANOVA, *F*_4,35_ = 4.012, *P* = 0.015). **l** SPT (2 day, one-way ANOVA, *F*_4,35_ = 3.872, *P* = 0.021). Data are shown as mean ± SEM. (*n* = 8). *P < 0.05, **P < 0.01. ANOVA analysis of variance, CA control antibody, FST forced swimming test, NA neutralizing antibody, N.S. not significant, R-KT (*R*)-ketamine, SB SB431542, SPT sucrose preference test, TST tail suspension test.

To study the role of TGF-β1 in the antidepressant effects of (*R*)-ketamine, we used two TGF-β receptor 1 inhibitors: RepSox and SB431542. Pretreatment with RepSox (10 mg/kg, i.p., 30 min) significantly blocked the antidepressant effects of (*R*)-ketamine in CSDS susceptible mice (Fig. 2a–d). Likewise, pretreatment with SB431542 (10 μM, 2 μl, i.c.v., 30 min) significantly blocked the antidepressant effects of (*R*)-ketamine in CSDS susceptible mice (Fig. 2e–h). Moreover, pretreatment with neutralizing antibody of TGF-β1 (1 μg/ml, 2 μL, i.c.v., 30 min) significantly blocked the antidepressant effects of (*R*)-ketamine in CSDS susceptible mice (Fig. 2i–l). These findings indicate that TGF-β1 might contribute to the antidepressant effects of (*R*)-ketamine in CSDS susceptible mice.

### Role of microglial TGF-β1

**Fig. 3:**
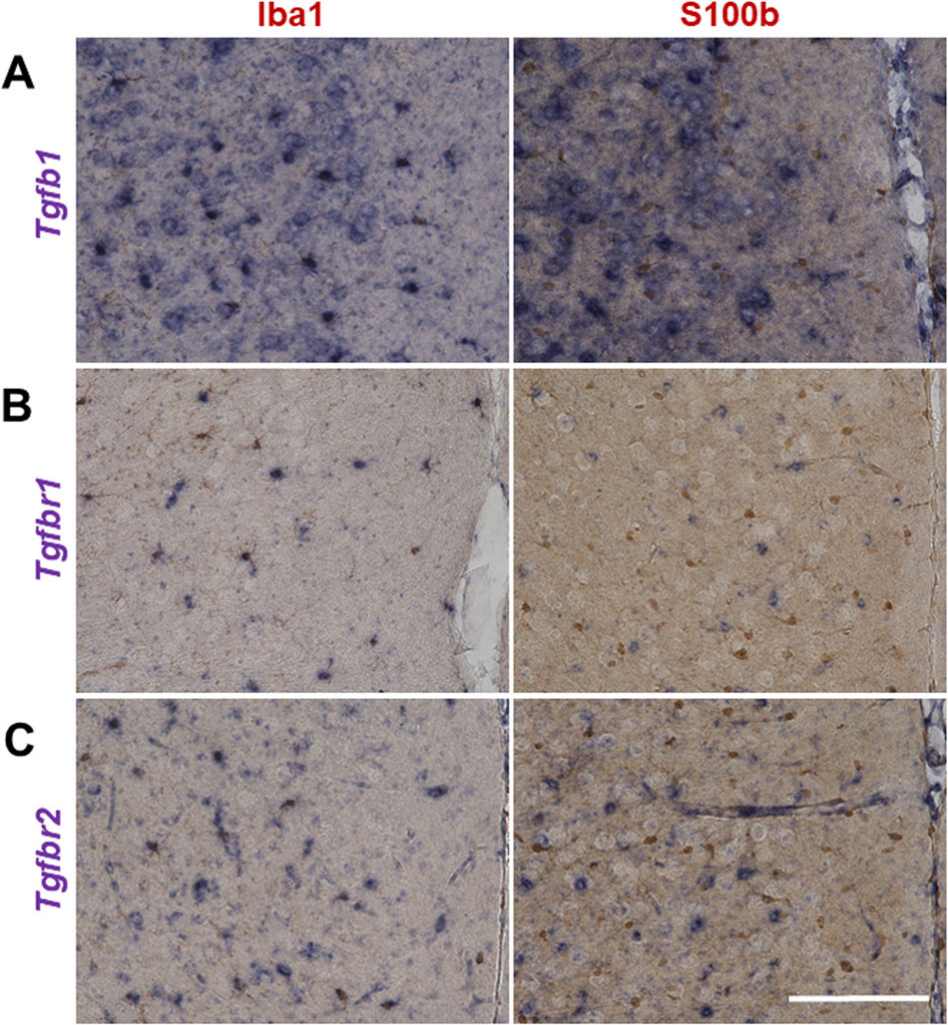
In situ hybridization and immunohistochemistry. **a** Representative image of *Tgfb1* mRNA (purple) and Iba1 protein (brown, marker for microglia) or S100b protein (brown, marker for astrocyte). **b** Representative image of *Tgfbr1* mRNA. **c** Representative image of *Tgfbr2* mRNA. *Tgfb1* and its receptors (*Tgfbr1* and *Tgfbr2*) are co-localized with microglia, but not astrocytes. Scale bar = 100 μm.

TGF-β1 is constitutively expressed in microglia into adulthood^55^. An earlier study demonstrated that TGF-β1 was necessary for the in vitro development of microglia and that microglia were absent in the brain of TGF-β1-deficient mice^56^, suggesting that TGF-β1 plays a key role in microglia. Microglia rely on cytokine signaling, such as activation of CSF1R and TGF-β1, for their survival^57^. In situ hybridization with cell-type marker immunostaining revealed high expression of *Tgfb1* and its receptors (*Tgfbr1* and *Tgfbr2*) in microglia, but not in astrocytes, in mouse brain PFC (Fig. 3).

**Fig. 4:**
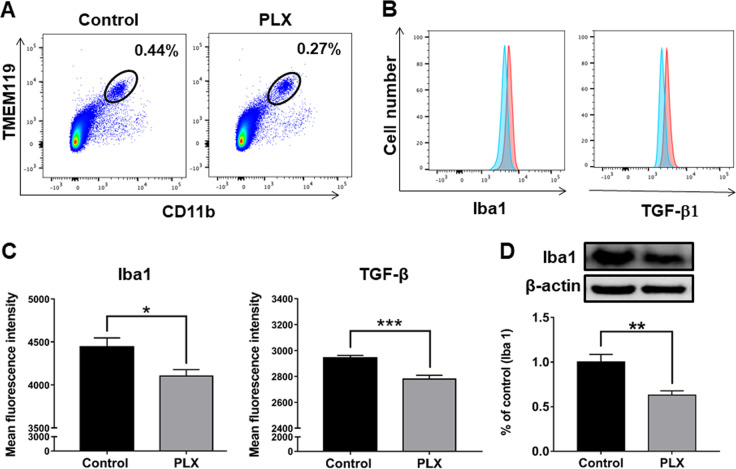
FACS analysis of PFC samples from vehicle or PLX3397 treated mice. **a** FACS analysis of CD11b-gated cells stained with antibody to TMEM119 in PFC samples of control mice and PLX3397 treated mice. **b** Iba1 and TGF-β1 expression in TMEM119^+^CD11b^+^ microglia were analyzed. Red histograms indicate control group and blue histograms indicate PLX3397 treated group. **c** The fluorescence intensity of both Iba1 and TGF-β1 in TMEM119^+^CD11b^+^ microglia in the PFC of PLX3397 treated mice was significantly (Iba1: *P* = 0.0186, TGF-β1: *P* = 0.0002) lower than that of control mice. **d** Western blot analysis of Iba1 in the PFC samples of control mice and PLX3397 treated mice. Representative bands of Western blot analysis. The expression of Iba1 in the PFC of PLX3397 treated mice was significantly (*P* = 0.0023) lower than that of control mice. Data are shown as mean ± SEM. (control group: *n* = 10, PLX group *n* = 9). *P < 0.05, **P < 0.01, ***P < 0.001.

To examine whether microglia TGF-β1 contributes to the antidepressant effects of (*R*)-ketamine, we studied the impact of microglial depletion on the antidepressant effects of (*R*)-ketamine. Preliminary experimentation revealed that i.c.v. injection of PLX3397, a potent CSF1R inhibitor, reduced the Iba1 protein in the mouse PFC (Fig. S2). In this study, we used the time (24 h) of PLX3397 (100 μM, 2 μl, i.c.v.). Using FACS analysis, we analyzed the expression of both Iba1 and TGF-β1 in TMEM119^+^CD11b^+^ microglia in the PFC. Pretreatment with PLX3397 significantly reduced the expression of both TGF-β1 and Iba1 in TMEM119^+^CD11b^+^ microglia (Fig. 4a–c). Furthermore, Western blot analysis revealed that PLX3397 injection reduced Iba1 protein in the PFC (Fig. 4d). These findings indicate partial depletion of microglia by PLX3397 in the PFC.

**Fig. 5:**
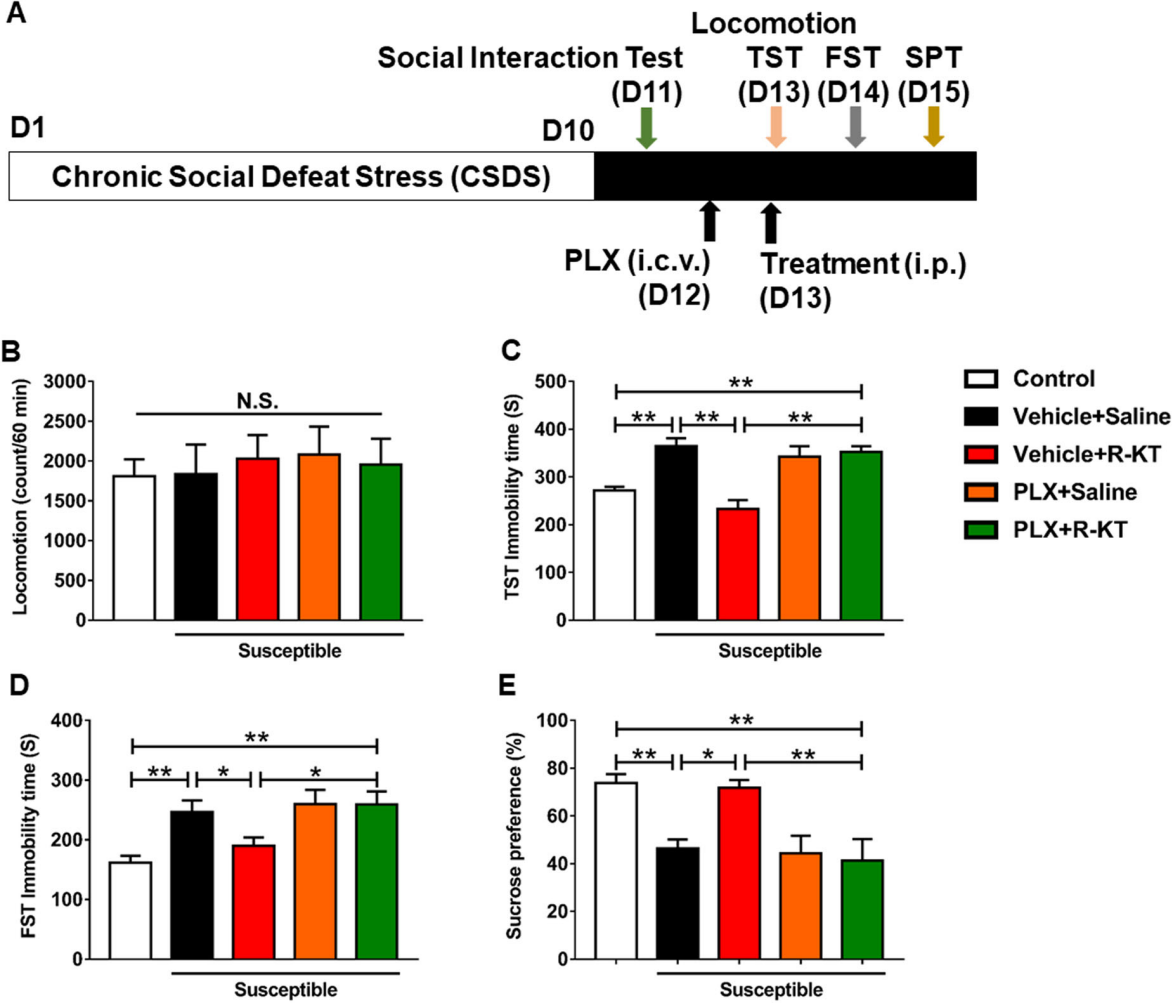
Effects of PLX3397 on antidepressant effects of (*R*)-ketamine in a CSDS model. **a** Chronic social defeat stress (CSDS) was performed from day 1 to day 10 for 10 days. Social interaction test was performed on day 11. On day 12, vehicle or PLX3397 was administered i.c.v. to CSDS susceptible mice. On day 13, saline or (*R*)-ketamine (10 mg/kg) was administered i.p. 24 h after injection of PLX3397. Locomotion and FST were performed 1 and 3 h after injection, respectively. FST and SPT were performed 1 and 2 days after injection, respectively. **b** Locomotion (1 h, one-way ANOVA, *F*_4,35_ = 0.226, *P* = 0.921). **c** TST (3 h, one-way ANOVA, *F*_4,35 _ = 13.706, *P* < 0.001). **d** FST (1 day, one-way ANOVA, *F*_4,35_ = 5.362, *P* = 0.005). **e** SPT (2 day, one-way ANOVA, *F*_4,35_ = 6.045, *P* = 0.003). Data are shown as mean ± SEM. (*n* = 8). *P < 0.05, **P < 0.01. ANOVA analysis of variance, FST forced swimming test, N.S. not significant, PLX PLX3397, R-KT (*R*)-ketamine, SPT sucrose preference test, TST tail suspension test.

Next, we studied the impact of PLX3397 on the antidepressant effects of (*R*)-ketamine in CSDS susceptible mice (Fig. 5a). There were no changes in locomotion among the five groups (Fig. 5b). Findings from the TST and the forced swim test (FST), showed that PLX3397 significantly blocked the antidepressant effects of (*R*)-ketamine for increased immobility time of both TST and FST (Fig. 5c, d). In the SPT, PLX3397 significantly blocked the effects of (*R*)-ketamine for reduced sucrose preference in CSDS susceptible mice (Fig. 5e). Collectively, partial depletion of microglia by PLX3397 significantly blocked the antidepressant effects of (*R*)-ketamine in CSDS susceptible mice (Fig. 5). These findings indicate that microglia-expressing molecules, including TGF-β1 and its receptors, contribute to the antidepressant effects of (*R*)-ketamine in a CSDS model.

### Antidepressant effects of TGF-β1 in rodent models of depression

**Fig. 6:**
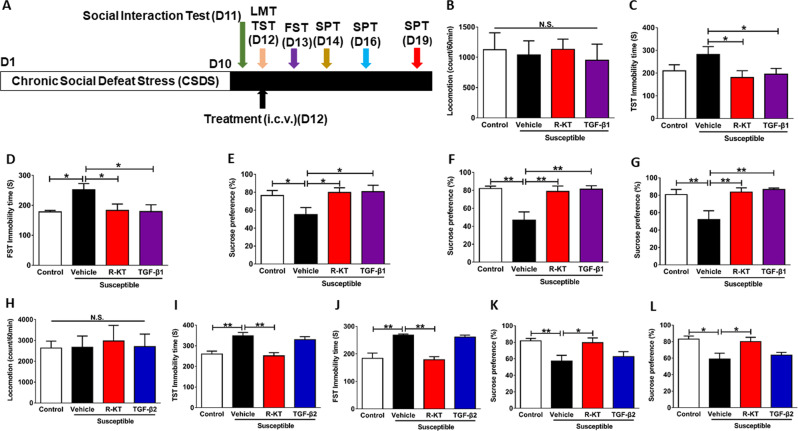
Effects of recombinant TGF-β1 and TGF-β2 in a CSDS model. **a** Chronic social defeat stress (CSDS) was performed from day 1 to day 10 for 10 days. Social interaction test was performed on day 11. On day 12, vehicle or TGF-β1 (or TGF-β2) was administered i.c.v. to CSDS susceptible mice. Locomotion and TST were performed 1 and 3 h after injection, respectively. FST was performed 1 day after injection. SPT was performed 2, 4, and 7 days after injection. **b** Locomotion (1 h, one-way ANOVA, *F*_3,20_ = 0.122, *P* = 0.946). **c** TST (3 h, one-way ANOVA, *F*_3,20_ = 2.352, *P* = 0.041). **d** FST (1 day, one-way ANOVA, *F*_3,20_ = 3.650, *P* = 0.030). **e** SPT (2 day, one-way ANOVA, *F*_3,20_ = 3.410, *P* = 0.037). **f** SPT (4 day, one-way ANOVA, *F*_3,20_ = 8.140, *P* = 0.001). **g** SPT (7 day, one-way ANOVA, *F*_3,20_ = 6.278, *P* = 0.004). **h** Locomotion (1 h, one-way ANOVA, *F*_3,20_ = 0.171, *P* = 0.975). **i** TST (3 h, one-way ANOVA, *F*_3,20_ = 10.093, *P* < 0.001). **j** FST (1 day, one-way ANOVA, *F*_3,20_ = 16.353, *P* < 0.001). **k** SPT (2 day, one-way ANOVA, *F*_3,20_ = 4.750, *P* = 0.012). **l** SPT (4 day, one-way ANOVA, *F*_3,20_ = 5.404, *P* = 0.007). Data are shown as mean ± SEM. (*n* = 6). *P < 0.05, **P < 0.01. ANOVA analysis of variance, FST forced swimming test, N.S. not significant, R-KT (*R*)-ketamine, SPT sucrose preference test, TST tail suspension test.

Finally, we studied whether mouse recombinant TGF-β1 has antidepressant effects in three animal models of depression. First, we studied the effects of TGF-β1 and TGF-β2 in the CSDS model (Fig. 6a). There were no changes in locomotion in the four groups (Fig. 6b, h). A single i.c.v. injection of (*R*)-ketamine (1 mg/ml, 2 μl) produced rapid and sustained antidepressant effects in CSDS susceptible mice, consistent with the previous report^58^. Similar to (*R*)-ketamine, i.c.v. infusion of TGF-β1 (10 ng/ml, 2 μl) significantly the increased immobility time of both TST and FST in CSDS susceptible mice (Fig. 6c, d). In the SPT, i.c.v. infusion of TGF-β1 significantly the reduced sucrose preference in CSDS susceptible mice (Fig. 6e-g). Interestingly, we detected the beneficial effects of TGF-β1 seven days after a single injection (Fig. 6g), indicating long-lasting antidepressant effects of TGF-β1. Conversely, TGF-β2 (10 ng/ml, 2 μl) did not produce antidepressant effects in CSDS susceptible mice, though (*R*)-ketamine (1 mg/ml, 2 μl) produced rapid and sustained antidepressant effects in the same model (Fig. 6h–l).

**Fig. 7:**
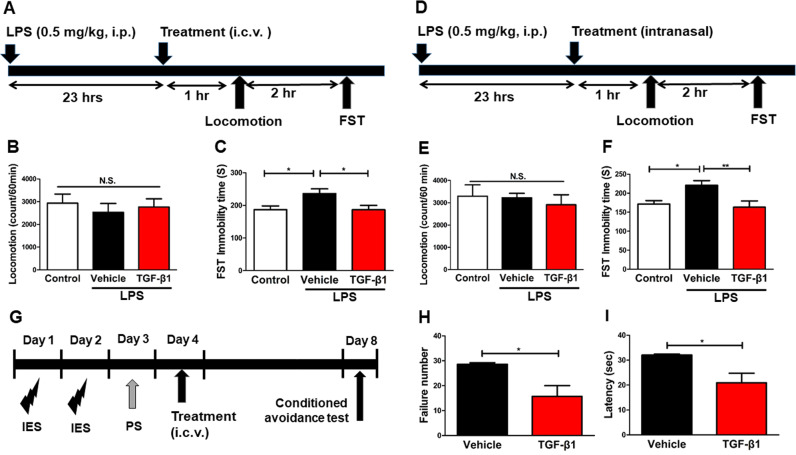
Effects of recombinant TGF-β1 in LPS model and LH model. **a** Saline or LPS (0.5 mg/kg) was administered i.p. to mice. Saline or TGF-β1 was administered i.c.v. to LPS-treated mice 23 h after LPS injection. Locomotion and FST were performed 1 and 3 h after injection, respectively. **b** Locomotion (1 h, one-way ANOVA, *F*_2,39_ = 0.122, *P* = 0.122). **c** FST (3 h, one-way ANOVA, *F*_2,39_ = 3.124, *P* = 0.045). Data are shown as mean ± SEM. (*n* = 14). *P < 0.05. **d** Saline or LPS (0.5 mg/kg) was administered i.p. to mice. Saline or TGF-β1 was administered intranasally to LPS-treated mice 23 h after LPS injection. Locomotion and FST were performed 1 and 3 h after injection, respectively. **e** Locomotion (1 h, one-way ANOVA, *F*_2,27_ = 0.255, *P* = 0.777). **f** FST (3 h, one-way ANOVA, F_2,27_ = 5.180, *P* = 0.013). Data are shown as mean ± SEM. (*n* = 10). *P < 0.05, **P < 0.01. **g** Rats received inescapable electric stress shock (IES) treatments on 2 days (day 1 and day 2), passed a post-shock test (PS) on day 3 to select learned helplessness (LH) rats with depression-like phenotype. On day 4, vehicle or TGF-β1 was administered i.c.v. into LH rats. On day 8 (4 days after i.c.v. injection), conditioned avoidance (CA) tests to study the antidepressant effect was performed. **h** The failure number of TGF-β1 treated LH rats was significantly (*P* = 0.0259) lower than that of vehicle treated LH rats. **i** The escape latency of TGF-β1 treated LH rats was significantly (*P* = 0.0281) lower than that of vehicle treated LH rats. Data are shown as mean ± SEM. (vehicle: *n* = 5, TGF-β1: *n* = 6). *P < 0.05. FST forced swimming test, N.S. not significant.

Moreover, a single i.c.v. infusion of TGF-β1 (10 ng/ml, 2 μl) significantly attenuated the increased immobility time of FST in LPS (0.5 mg/kg)-treated mice (Fig. 7a–c). In addition, a single intranasal administration of TGF-β1 (1.5 μg, 15 μl) significantly attenuated the increased immobility time of FST in LPS-treated mice (Fig. 7d–f). In a rat LH model, bilateral i.c.v. infusion of TGF-β1 (250 ng/side) significantly reduced the failure number and latency of LH rats 4 days after i.c.v. injection (Fig. 7g–i). These findings indicate that recombinant TGF-β1 has ketamine-like robust antidepressant effects in rodent models of depression.

## Discussion

The main findings of this study are as follows: First, RNA-sequencing and GSEA revealed the role of TGF-β signaling in the beneficial antidepressant effects of (*R*)-ketamine compared with (*S*)-ketamine. RT-PCR revealed reduced expression of *Tgfb1* and its receptors (*Tgfbr1* and *Tgfbr2*) in the PFC and the hippocampus from CSDS susceptible mice. Furthermore, (*R*)-ketamine, but not (*S*)-ketamine, attenuated the reduced expression of these genes in the PFC and the hippocampus of CSDS susceptible mice. Second, pharmacological inhibitors and neutralizing antibody of TGF-β1 blocked the antidepressant effects of (*R*)-ketamine in CSDS susceptible mice, indicating a role of TGF-β1 signaling in the antidepressant effects of (*R*)-ketamine. Third, partial depletion of microglia by PLX3397 blocked antidepressant effects of (*R*)-ketamine in CSDS susceptible mice, indicating a role of microglia in the antidepressant effects of (*R*)-ketamine. Lastly, recombinant TGF-β1 elicited rapid-acting and long-lasting antidepressant effects in CSDS, LPS, and LH models of depression. Overall, it appears likely that (*R*)-ketamine can exert antidepressant effects by normalizing microglial TGF-β1 signaling in the PFC and the hippocampus of CSDS susceptible mice. Furthermore, TGF-β1 has ketamine-like antidepressant effects in rodent models.

Microglia are the only cell type that express CSF1R. CSF1R knockout mice are devoid of microglia^59^. Moreover, it has been reported that repeated treatment with CSF1R inhibitors, such as PLX3397, cause a dramatic reduction in the number of microglia within the adult brain^48–50^. Interestingly, microglia are absent in the brains of central nervous system TGF-β1 knockout mice^56^. Thus, microglia in the adult brain are physiologically dependent upon CSF1R and TGF-β1 signaling^57^. In this study, a single i.c.v. injection of PLX3397 produced significant reduction of Iba1 and TGF-β1 in the PFC, suggesting partial depletion of microglia in the PFC. Interestingly, pretreatment of PLX3397 significantly blocked the antidepressant effects of (*R*)-ketamine in CSDS susceptible mice. Overall, it appears likely that microglial TGF-β1 in the PFC might contribute to the antidepressant effects of (*R*)-ketamine.

In this study, i.c.v. infusion of TGF-β1 produced rapid-acting and long-lasting antidepressant effects in a CSDS model, an LPS-induced model, and an LH model. Notably, we detected the antidepressant effects of TGF-β1 in a CSDS model and an LH model 7 days and 4 days after a single dose, respectively. Collectively, the antidepressant effects of TGF-β1 in these models are similar to those of (*R*)-ketamine, suggesting that TGF-β1 has (*R*)-ketamine-like long-lasting antidepressant effects. Taylor et al^60^. showed that a single i.c.v. injection of TGF-β1 4 h after intracerebral hemorrhage (ICH) produced complete recovery of motor function at 24 h, and that this recovery persisted for at least one week. Furthermore, i.c.v. injection of TGF-β1 alleviated *N*-methyl-4-phenylpyridinium ion (MPP^+^)-induced microglial inflammatory response and dopaminergic neuronal loss in the substantia nigra, indicating that TGF-β1 plays a role in the pathology of Parkinson’s disease (PD). Collectively, it is possible that TGF-β1 can produce rapid and long-lasting beneficial effects in several models, such as depression, ICH, and PD.

Notably, intranasal administration of TGF-β1 has rapid-acting antidepressant effects in LPS-treated mice. A previous study showed that intranasal administration of TGF-β1 ameliorated neurodegeneration in the mouse brain after β-amyloid_1–42_ injection^44^. It has also been reported that TGF-β1 administered intranasally entered several brain regions, such as the PFC and the hippocampus, of control adult mice, whereas no increase was observed in the blood and peripheral organs^61^, indicating good permeability of the blood brain barrier for TGF-β1. It is also reported that CSDS alters blood brain barrier integrity through loss of tight junction protein Cldn5^62^. In addition, TGF-β1 might be free of the psychotomimetic side-effects of ketamine and its potential for abuse in humans, as TGF-β1 does not interact with NMDAR in the brain. Therefore, it is likely that intranasal administration of TGF-β1 would be a novel potential therapeutic approach for depression.

This study has some limitations. In this study, we used the CSF1R inhibitor to delete microglia in the brain although the partial depletion of microglia was detected. It is of great interest to investigate the role of microglia in the antidepressant effects of (*R*)-ketamine using CSF1R knockout mice since CSF1R knockout mice are devoid of microglia^59^. Furthermore, it is also of interest to investigate the role of microglial TGF-β1 in the antidepressant effects of (*R*)-ketamine using TGF-β1 knockout mice since microglia were absent in the brain of TGF-β1 knockout mice^56^.

In conclusion, this study shows that TGF-β1 in the microglia might contribute to the antidepressant effects of (*R*)-ketamine in animal models of depression. Furthermore, similar to (*R*)-ketamine, TGF-β1 seems to rapid-acting and long-lasting antidepressant effects. Therefore, it is likely that TGF-β1 would be a new rapid-acting and sustained antidepressant.

## Supplementary information

Supplemental information
