# Supplementary material for: Essential role of microglial transforming growth factor-β1 in antidepressant actions of (R)-ketamine and the novel antidepressant TGF-β1
Source: Transl Psychiatry. 2020 Jan 27;10:32. doi: 10.1038/s41398-020-0733-x (PMC7026089; doi:10.1038/s41398-020-0733-x)
Supplement: Supplementary file 1 — Supplemental information [file 41398_2020_733_MOESM1_ESM.docx]

**Supplemental Information**

**Essential role of microglial transforming growth factor-β1 in antidepressant actions of (*R*)-ketamine and the novel antidepressant TGF-β1**

Kai Zhang, Chun Yang, Lijia Chang, Akemi Sakamoto, Toru Suzuki, Yuko Fujita, Youge Qu, Siming Wang, Yaoyu Pu, Yunfei Tan, Xingming Wang, Tamaki Ishima, Yukihiko Shirayama, Masahiko Hatano, Kenji F. Tanaka and Kenji Hashimoto

Division of Clinical Neuroscience (KZ, CY, LC, YF, YQ, SW, YP, YT, XW, TI, YS, KH), Chiba University Center for Forensic Mental Health, Chiba 260-8670, Japan; Department of Biomedical Science (AS, MH), Chiba University Graduate School of Medicine, Chiba 260-8670, Japan, Department of Neuropsychiatry (TS, KFT), Keio University School of Medicine, Tokyo 160-8585, Japan, and Department of Psychiatry (YS), Teikyo University Chiba Medical Center, Chiba 299-0111, Japan

**Correspondence:** Dr. Kenji Hashimoto. Division of Clinical Neuroscience, Chiba University Center for Forensic Mental Health, 1-8-1 Inohana, Chiba 260-8670, Japan. E-mail: hashimoto@faculty.chiba-u.jp

**Short title:** TGF-β1 in the antidepressant actions of (*R*)-ketamine

**Supplemental methods**

**Chronic social defeat stress (CSDS) model**

Every day the C57BL/6 mice were exposed to a different CD1 aggressor mouse for 10 min, total for 10 days. When the social defeat session ended, the resident CD1 mouse and the intruder mouse were housed in one half of the cage separated by a perforated Plexiglas divider to allow visual, olfactory, and auditory contact for the remainder of the 24-h period. At 24 h after the last session, all mice were housed individually. On day 11, a social interaction test (SIT) was performed to identify subgroups of mice that were susceptible and unsusceptible to social defeat stress. This was accomplished by placing mice in an interaction test box (42×42 cm) with an empty wire-mesh cage (10×4.5 cm) located at one end. The movement of the mice was tracked for 2.5 min, followed by 2.5 min in the presence of an unfamiliar aggressor confined in the wire-mesh cage. The duration of the subject’s presence in the “interaction zone” (defined as the 8-cm-wide area surrounding the wiremesh cage) was recorded by a stopwatch. The interaction ratio was calculated as time spent in an interaction zone with an aggressor / time spent in an interaction zone without an aggressor. An interaction ratio of 1 was set as the cutoff: mice with scores < 1 were defined as “susceptible” to social defeat stress and those with scores ≥ 1 were defined as “unsusceptible”. Approximately 70 - 80 % of mice were susceptible after CSDS. Susceptible mice were randomly divided in the subsequent experiments. Control mice without social defeat stress were housed in the same cage before the behavioral tests.

For experiments of pharmacological inhibition, RepSox (10 mg/kg, i.p.) was administered 30 min before i.p. administration of (*R*)-ketamine (10 mg/kg) in CSDS susceptible mice. Under isoflurane anesthesia, SB431542 (10 μM, 2 μL, i.c.v.) or neutralized TGF-β1-3 antibody (1 μg/ml, 2 μL, i.c.v.) was administered 30 min before i.p. administration of (*R*)-ketamine (10 mg/kg) in CSDS susceptible mice. Subsequently, behavioral tests were performed.

**Gene expression analysis by quantitative real-time PCR**

A quantitative RT-PCR system (Step One Plus, Thermo Fisher Scientific, Yokohama, Japan) was used to measure mRNAs. The specific mRNA transcripts were quantified by TaqMan Gene Expression assays (Thermo Fisher Scientific, Yokohama, Japan). Expression levels of *Tgfb1* (Mm01178820_m1), *Tgfb2* (Mm00436955_m1), *Tgfbr1* (Mm00436964_m1), and *Tgfbr2* (Mm03024091_m1) were measured. Total RNA was extracted by use of an RNeasy Mini Kit (Qiagen, Hilden, Germany). The purity of total RNA was assessed by Bio photometer plus (Eppendorf, Hamburg, Germany). The RNA samples were used in the first strand cDNA synthesis with High Capacity cDNA Reverse Transcription Kit (#4368813 Thermo Fisher Scientific, Yokohama, Japan). All samples were tested in triplicate and average values were used for quantification. The average values were normalized to Vic-labeled *Actb* mRNA (#4352341E: pre-developed TaqMan Assay Reagents, Thermo Fisher Scientific, Yokohama, Japan).

**Behavioral tests**

Behavioral tests, including locomotion, tail suspension test (TST), forced swimming test (FST) and 1% sucrose preference test (SPT), were performed.

**Locomotion**: The locomotor activity was measured by an animal movement analysis system SCANETMV-40 (MELQUEST Co., Ltd., Toyama, Japan). The mice were placed in experimental cages (length × width × height: 560 × 560 × 330 mm). The cumulative exercise was recorded for 60 minutes. Cages were cleaned between testing session.

**TST**: A small piece of adhesive tape placed approximately 2 cm from the tip of the tail for mouse. A single hole was punched in the tape and mice were hung individually, on a hook. The immobility time was recorded for 10 minutes. Mice were considered immobile only when they hung passively and completely motionless.

**FST:** The FST was tested by an automated forced-swim apparatus SCANETMV-40 (MELQUEST Co., Ltd., Toyama, Japan). The mice were placed individually in a cylinder (diameter: 23 cm; height: 31 cm) containing 15 cm of water, maintained at 23 ± 1℃. Immobility time from activity time as (total) – (active) time was calculated by the apparatus analysis software. The immobility time for mouse was recorded for 6 minutes.

**SPT**: Mice were exposed to water and 1% sucrose solution for 48 h, followed by 4 hours of water and food deprivation and a 1 hour exposure to two identical bottles, one is water, and another is 1% sucrose solution. The bottles containing water and sucrose were weighed before and at the end of this period. The sucrose preference was calculated as a percentage of sucrose solution consumption to the total liquid consumption.

**Learned helplessness (LH) model**

To create an LH paradigm, the animals are initially exposed to uncontrollable stress. When the animal is later placed in a situation where the shock is controllable (escapable), the animal not only fails to acquire the escape response, but also often makes no efforts to escape the shock at all. The LH behavioral tests were performed using the Gemini Avoidance System (San Diego Instruments, San Diego, CA). This apparatus is divided into two compartments by a retractable door. On days 1 and 2, the rats were subjected to 30 inescapable electric foot-shocks (0.65 mA, 30-s duration, administered at random intervals averaging 18-42 s). On day 3, a two-way conditioned avoidance test was performed as a post-shock test to determine whether the rats would exhibit the predicted escape deficits. This screening session consisted of 30 trials in which electric foot-shocks (0.65 mA, 6-s duration, administered at random intervals with a mean of 30 s) were preceded by a 3-s conditioned stimulus tone that remained on until the shock was terminated. Rats with more than 25 escape failures among the 30 trials were regarded as having reached the LH criterion and were used in further experiments. Approximately 65% of the rats met this criterion.

On day 4, the LH rats received bilateral injection of saline (2.5 μl/side, i.c.v.) or TGF-β1 (100 ng/μl, 2.5 μl/side, 250 ng/side, i.c.v.) under anesthesia with isoflurane and sodium pentobarbital. On day 8 (4 days after a single injection), a two-way conditioned avoidance test was performed.

**Western blot analysis of Iba1**

Mice were killed by cervical dislocation and brains were rapidly removed from the skull. PFC was dissected on ice, and stored at −80°C. Tissue samples were homogenized in Laemmli lysis buffer. Aliquots (20 μg) of protein were measured using the DC protein assay kit (Bio-Rad), and incubated for 5 min at 95°C,with an equal volume of 125mM Tris-HCl, pH 6.8, 20% glycerol, 0.1% bromophenol blue, 10% β-mercaptoethanol, 4% SDS, and subjected to SDS polyacrylamide gel electrophoresis using AnyKD minigels (Mini-PROTEAN TGX Precast Gel; BioRad). Proteins were transferred onto PVDF membranes using a Trans Blot Mini Cell (Bio-Rad). For immunodetection, the blots were blocked with 2% BSA in TBST (TBS + 0.1% Tween-20) for 1 h at room temperature, and kept with primary antibodies overnight at 4 °C. The following primary antibodies were used: Iba1 (1:1000; Code No. 016-20001: Wako Pure Chemical Industries, Ltd., Tokyo, Japan). The next day, blots were washed three times in TBST, and incubated with horseradish peroxidase-conjugated anti-rabbit antibody (1:10,000) 1 h, at room temperature. After a final three washes with TBST, bands were detected using enhanced chemiluminescence (ECL) plus the Western Blotting Detection system (GE Healthcare Bioscience). The blots were then washed three times in TBST and incubated with the primary antibody directed against β-actin (1:10,000; Sigma-Aldrich). Images were captured with a Fuji LAS3000-mini imaging system (Fujifilm, Tokyo, Japan), and immunoreactive bands were quantified.


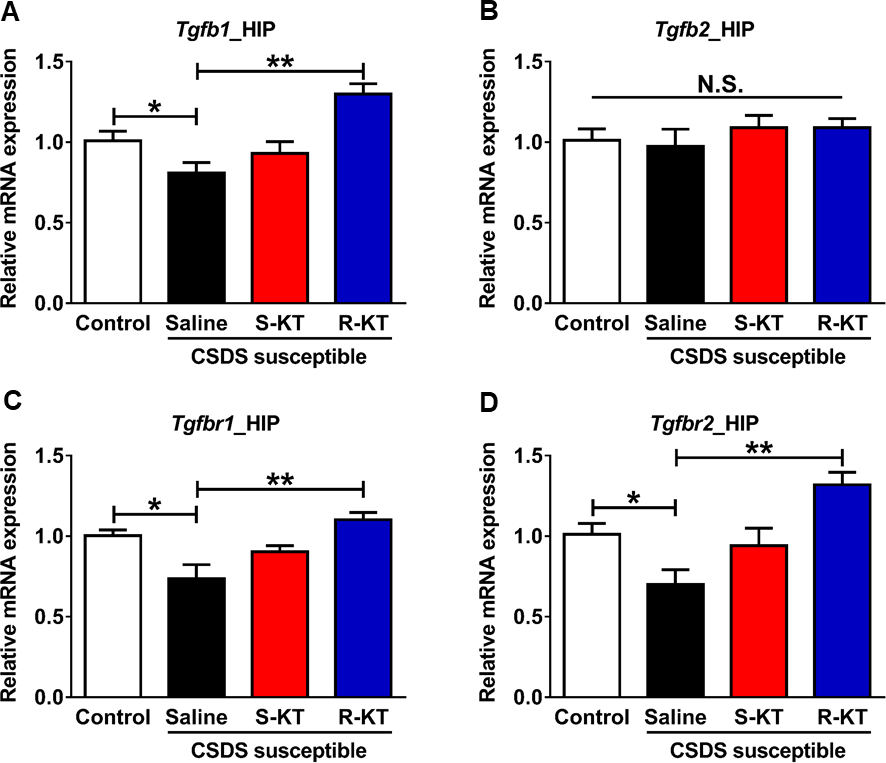


**Figure S1. Effects of ketamine enantiomers on gene expression of *Tgfb1* and its receptors (*Tgfbr1* and *Tgfbr2*) in the hippocampus of CSDS susceptible mice**

(A): *Tgfb1* mRNA in the hippocampus (one-way ANOVA, F_3,20_ = 10.136, P < 0.001). (B): *Tgfb2* mRNA in the hippocampus (one-way ANOVA, F_3,20_ = 0.530, P = 0.667). (C): *Tgfbr1* mRNA in the hippocampus (one-way ANOVA, F_3,20_ = 7.989, P = 0.001). (D): *Tgfbr2* mRNA in the hippocampus (one-way ANOVA, F_3,20_ = 8.443, P = 0.001). Data are shown as mean ± SEM. (n = 6). *P < 0.05, **P < 0.01. ANOVA, analysis of variance; NS, not significant; R-KT, (*R*)-ketamine; S-KT, (*S*)-ketamine.


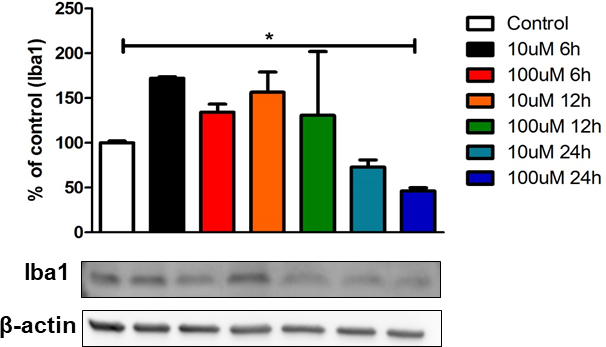


**Figure S2. Western blot of Iba1 in the PFC after a single i.c.v. injection of PLX3397**

The samples of PFC were collected after single i.c.v. injection of PLX3397. Western blot analysis of Iba1 in the PFC was performed. Data are shown as mean ± SEM. (n = 3). *P < 0.05 compared to control group.
